# Thalamocortical Connectivity Predicts Cognition in Children Born Preterm

**DOI:** 10.1093/cercor/bhu331

**Published:** 2015-01-16

**Authors:** Gareth Ball, Libuse Pazderova, Andrew Chew, Nora Tusor, Nazakat Merchant, Tomoki Arichi, Joanna M. Allsop, Frances M. Cowan, A. David Edwards, Serena J. Counsell

**Affiliations:** 1 Centre for the Developing Brain, Division of Imaging Sciences & Biomedical Engineering, King's College London, St Thomas’ Hospital, SE1 7EH, UK; 2 Department of Paediatrics, Imperial College London, Hammersmith Hospital, W12 0HS, UK

**Keywords:** brain, cognition, diffusion magnetic resonance imaging, infant, preterm

## Abstract

Thalamocortical connections are: essential for brain function, established early in development, and significantly impaired following preterm birth. Impaired cognitive abilities in preterm infants may be related to disruptions in thalamocortical connectivity. The aim of this study was to test the hypothesis: *thalamocortical connectivity in the preterm brain at term-equivalent is correlated with cognitive performance in early childhood*. We examined 57 infants who were born <35 weeks gestational age (GA) and had no evidence of focal abnormality on magnetic resonance imaging (MRI). Infants underwent diffusion MRI at term and cognitive performance at 2 years was assessed using the Bayley III scales of Infant and Toddler development. Cognitive scores at 2 years were correlated with structural connectivity between the thalamus and extensive cortical regions at term. Mean thalamocortical connectivity across the whole cortex explained 11% of the variance in cognitive scores at 2 years. The inclusion of GA at birth and parental socioeconomic group in the model explained 30% of the variance in subsequent cognitive performance. Identifying impairments in thalamocortical connectivity as early as term equivalent can help identify those infants at risk of subsequent cognitive delay and may be useful to assess efficacy of potential treatments at an early age.

## Introduction

The incidence of preterm birth is increasing and it is well recognized that preterm birth can lead to a range of neurologic and cognitive disorders. These disorders impart a significant morbidity and adversely affect performance at school and into adult life. Cognitive delay associated with preterm birth now affects a large number of children (Marlow et al. 2005; Delobel-Ayoub et al. 2009; Johnson et al. 2009) and represents a tremendous emotional, social, and economic burden for their families and society. Identifying infants at risk of cognitive deficit is crucial as therapies aimed at treating preterm brain injury are emerging and methods that are able to assess efficacy of potential treatments at an early stage are required (Rees et al. 2011; Bain et al. 2012). Validated objective measures that relate to subsequent cognitive function will facilitate the translation of treatment trials in to clinical practice. The specific neuroanatomic correlates of cognitive impairment in preterm infants remain unclear, but may be due to the joint disruption of the developing thalamocortical system in the preterm brain (Ball et al. 2012; Ball, Boardman et al. 2013).

The time between preterm birth and term equivalent age represents a critical period for the establishment of functional thalamocortical connections (Kostovic et al. 2002; Kostovic et al. 2014). Thalamic afferents project toward the cortex and form transient, functional circuits with subplate neurons by mid-gestation, before proliferating into the cortical layers. During this process, key cell populations in both white and gray matter are particularly vulnerable. Subplate neurons, thalamic neurons, and pre-myelinating oligodendrocytes are susceptible to injury from a number of causes including hypoxia/ischemia and infection/inflammation, all of which are more common during the preterm period (Ghosh and Shatz 1993; Agresti et al. 1996; McQuillen et al. 2003; Back et al. 2005; Ligam et al. 2009). Damage to one or all of these cell populations during this critical phase could impact on the microstructural development of the cortex, thalamus, and connective white matter fibers (Ghosh et al. 1990; Pierson et al. 2007; Volpe 2009a; Dean et al. 2013). Preoligodendrocytes, which are the predominant white matter cell type in the preterm brain (Back et al. 2001), are specifically sensitive to downstream pathogenic mechanisms including microglial activation, excitotoxicity, and free radical attack (Back et al. 1998; Haynes et al. 2005; Billiards et al. 2006; Verney et al. 2010) and axons that are preparing to myelinate are more sensitive to hypoxic injury than neighboring axons (Alix et al. 2012).

The concurrent timing of the injurious processes and thalamocortical development may contribute to the aberrant brain growth that has been observed following preterm birth with magnetic resonance (MR) imaging (Ajayi-Obe et al. 2000; Boardman et al. 2006; Kapellou et al. 2006; Anjari et al. 2007; Rathbone et al. 2011; Smith et al. 2011; Ball et al. 2012; Ball, Srinivasan et al. 2013; Dean et al. 2013; Vinall et al. 2013; Pandit et al. 2014). Indeed, microstructural alterations in cortical and subcortical gray matter alongside disrupted white matter connections may reflect developmental impairments of the thalamocortical unit as a whole by term-equivalent age (Ball et al. 2012; Ball, Boardman et al. 2013; Ball, Srinivasan et al. 2013).

Recent neuroimaging studies in adults have highlighted the impact of alterations in thalamocortical neural networks on cognitive function (Charlton et al. 2010; Hughes et al. 2012). The aim of this study was to test the hypothesis *thalamocortical connectivity in the preterm brain at term equivalent age is correlated with cognitive performance in early childhood*.

## Materials and Methods

Permission for this study was granted by Queen Charlotte's and Hammersmith Hospitals Research Ethics Committee (07/H0704/99) and written parental consent was acquired prior to imaging.

Inclusion criteria were preterm birth <35 weeks gestational age (GA), MR with diffusion MR imaging (d-MRI) at around term equivalent age, no evidence of focal abnormality on conventional MR imaging and neurodevelopmental assessment at 2 years corrected age. A total of 102 preterm infants underwent MRI at term equivalent age between October 2007 and July 2010. Of these 8 infants were excluded due to motion artefact on d-MRI or MRI, 18 infants were excluded due to focal abnormality on MR imaging (cystic periventricular leukomalacia, *n* = 7; unilateral periventricular hemorrhagic infarction, *n* = 7, cerebellar hemorrhage, *n* = 3; post-hemorrhagic ventricular dilatation, *n* = 1), 11 underwent MRI but d-MRI was not acquired and 8 infants (including 3 sets of twins) did not attend for follow-up examination. The final study population therefore consisted of 57 children (29 males) who were born at a median GA of 29^+5^ (25^+5^–34^+4^) weeks and scanned at 40^+6^ (38–46) weeks post-menstrual age (PMA). Parental socioeconomic status (SES) was categorized according to a standard occupational classification (Office of National Statistics 1991), which ranges from 1–6, where 1 represents higher managerial and professional occupations and 6 represents never worked or long-term unemployed. SES was derived from the parent whose occupation was graded the highest. The characteristics of the infants are described in Table 1. Eleven infants were included in a previous study of thalamocortical connectivity in preterm infants (Ball, Boardman et al. 2013).

**Table 1:** Infant characteristics

Characteristic	Value
Median (range) GA at birth (weeks)	29^+5^ (25^+5^–34^+4^)
Median (range) birthweight (grams)	1210 (560–2280)
Male, no (%)	25 (44%)
Small for gestational age^a^, no (%)	10 (17%)
Chronic lung disease^b^, no (%)	9 (15%)
Received a full course of antenatal steroids, no (%)	44 (77%)
Culture positive post-natal sepsis, no (%)	7 (12%)
Mean (± SD) parental SES	2.4 (±1.4)

^a^Defined as <10th percentile.

^b^Defined as requirement for supplementary oxygen at 36 weeks PMA.

### MR Imaging

MR imaging was performed on a 3-T MR system sited on the neonatal intensive care unit.

*T*_1_- and *T*_2_-weighted MR imaging and single shot echo planar d-MRI were acquired using an 8-channel phased array head coil. Pulse sequence parameters were as follows; *T*_1_-weighted MR imaging; repetition time (TR) = 17 ms, echo time (TE) = 4.6 ms, flip angle 13°, slice thickness 0.8 mm, field-of-view 210 mm, matrix 256 × 256 (voxel size: 0.82 × 0.82 × 0.8). *T*_2_ weighted fast-spin echo MR imaging; TR = 8670 ms, TE = 160 ms, flip angle 90°, slice thickness 1 mm, field-of-view 220 mm, matrix 256 × 256 (voxel size: 0.86 × 0.86 × 1). d-MRI was acquired in the transverse plane in 32 non-collinear directions using the following parameters; TR = 8000 ms, TE = 49 ms, slice thickness 2 mm, field-of-view 224 mm, matrix 128 × 128 (voxel size: 1.75 × 1.75 × 2 mm), *b*-value: 750 s/mm^2^, SENSE factor of 2.

All examinations were supervised by a pediatrician experienced in MR imaging procedures. Infants were sedated with oral chloral hydrate (25–50 mg/kg) prior to scanning and pulse oximetry, temperature, and electrocardiography data were monitored throughout. Ear protection was used, comprising earplugs molded from a silicone-based putty (President Putty, Coltene Whaledent, Mahwah, NJ, USA) placed in the external auditory meatus and neonatal earmuffs (MiniMuffs, Natus Medical Inc., San Carlos, CA, USA).

### Assessment of Thalamocortical Connectivity

Thalamocortical connectivity was defined using methods described previously. Briefly, cortical gray matter masks were derived from *T*_2_-weighted images using age-specific tissue probability priors (Serag et al. 2012). Each mask was parcellated into a set of ∼1000 (500 per hemisphere) randomly distributed and equally spaced target regions of similar volume using Poisson disk sampling (Cook 1986; Bridson 2007). Cortical hemispheres were parcellated separately to ensure that no target regions incorporated voxels from both hemispheres. Each subject's cortical mask was parcellated 25 times allowing thalamocortical connectivity to be mapped iteratively, producing a voxel-wise map of connectivity that is not dependent on the accurate delineation of corresponding cortical regions.

For probabilistic tractography, all cortical parcellations were transformed from *T*_2_-space into diffusion space using the IRTK software package (Rueckert et al. 2003); (www.doc.ic.ac.uk/~dr/software/). d-MRI data were pre-processed using FSL's Diffusion Toolkit (FDT; www.fmrib.ox.ac.uk/fsl/). After eddy-current correction, BedpostX was used to fit a 2-compartment partial volume model of diffusion (Behrens et al. 2003, 2007). For each set of cortical target regions, streamlines were propagated from a thalamic seed mask defined using a population-average template according to previously described anatomical border (Srinivasan et al. 2007; Ball, Boardman et al. 2013). Around 500 000 streamlines were propagated from each thalamic seed mask (one mask per cerebral hemisphere), tracking stopped when streamlines reached a target region, left the brain mask, entered voxels containing cerebrospinal fluid (CSF), breached a curvature threshold or crossed into the contralateral hemisphere.

Thalamocortical connectivity was defined along the length of all streamlines connecting the thalamus to a given cortical target region using a modified version of the ProbtrackX algorithm. This approach incorporates information from diffusional anisotropy at each voxel and fiber orientation of pathways traced between remote regions to give an estimate of white matter microstructure specific to thalamocortical tracts. (Behrens et al. 2007; Iturria-Medina et al. 2008; Robinson et al. 2010; Ball, Boardman et al. 2013). Briefly, orientation distribution functions (ODF) were calculated from the partial volume model of diffusion at each voxel. As streamlines pass between adjacent voxels, the overlap between ODFs approximates the probability of diffusive transfer between voxels. Mean anisotropy, calculated by averaging the diffusive transfer between adjacent voxels connected by a streamline and multiplied by the number of times each voxel was sampled during tractography, was integrated along the length of all streamlines that reached a cortical target region as a surrogate measure of structural connectivity (Robinson et al. 2010). This value was then mapped onto each target region to create a cortical map of connectivity. By performing tractography multiple times between the thalamus and the full set of randomly generated target regions, a distribution of connectivity estimates was defined on a per-voxel basis and used to create a voxel-wise map of thalamocortical connectivity across the whole cortex.

For statistical analysis, thalamocortical connectivity maps were transformed into a common reference space. Each *T*_2_-weighted image was aligned to a population-average neonatal template using nonlinear registration (Serag et al. 2012). Each infant's thalamocortical connectivity maps were transformed into the reference space alongside gray matter tissue probability maps. In order to mitigate the effects of misalignment from registration, intersubject variability and partial volume contamination, a recently developed technique (Gray-matter Based Spatial Statistics, derived from Tract Based Spatial Statistics) was used to project cortical data onto a skeletonized representation of group mean cortical anatomy for statistical analysis (Ball, Srinivasan et al. 2013).

### Assessment of Diffusivity in Cortical and Thalamic Gray Matter

In order to extend the analysis to the whole of the thalamocortical system: microstructural development of the cortex and thalamus was assessed using voxel-wise measures of mean diffusivity in the cortical gray matter and mean diffusivity across the thalamus extracted from the cortical skeleton and thalamic masks, respectively.

### Statistical Analysis

Voxel-wise statistical analysis of mean thalamocortical connectivity and cortical diffusivity was performed with FSL's Randomise (v2.5). All statistical images were subject to family wise error correction for multiple comparisons after Threshold-Free Cluster Enhancement (TFCE) (Smith and Nichols 2009). Linear regression was performed to assess the relationship between thalamocortical connectivity and mean diffusivity in cortex and thalamus at term and cognitive scores at 2 years, adjusted for GA at birth, age at scan and parental SES. Statistical analysis was performed with SPSS 21.0 (SPSS Inc., Chicago, IL, USA).

### Cognitive Scores

Children were assessed using the Bayley Scales of Infant and Toddler Development III (Bayley 2006) at a median (range) of 24.5 (22.5–27) months corrected age. The Bayley III comprises 5 scales: Cognitive, Language (Expressive and Receptive) and Motor (Gross and Fine). This study assessed specifically the relationship between cognitive scores and thalamocortical connectivity. In addition, the children underwent a neurological examination and evidence of cerebral palsy was specifically looked for (Bax et al. 2005). All assessments were carried out by developmental pediatricians experienced in assessing preterm infants of this age, and unaware of the quantitative MRI findings.

## Results

### Cognitive Scores

The mean (±standard deviation) of the BSID-III cognitive scaled scores for the children was 9.73 ± 2.5 and the cognitive composite scores derived from the scaled scores was 98 ± 14. None of the children had cerebral palsy or major sensory deficits.

### Correlation Between Thalamocortical Connectivity and Cognition

**Figure 1.**
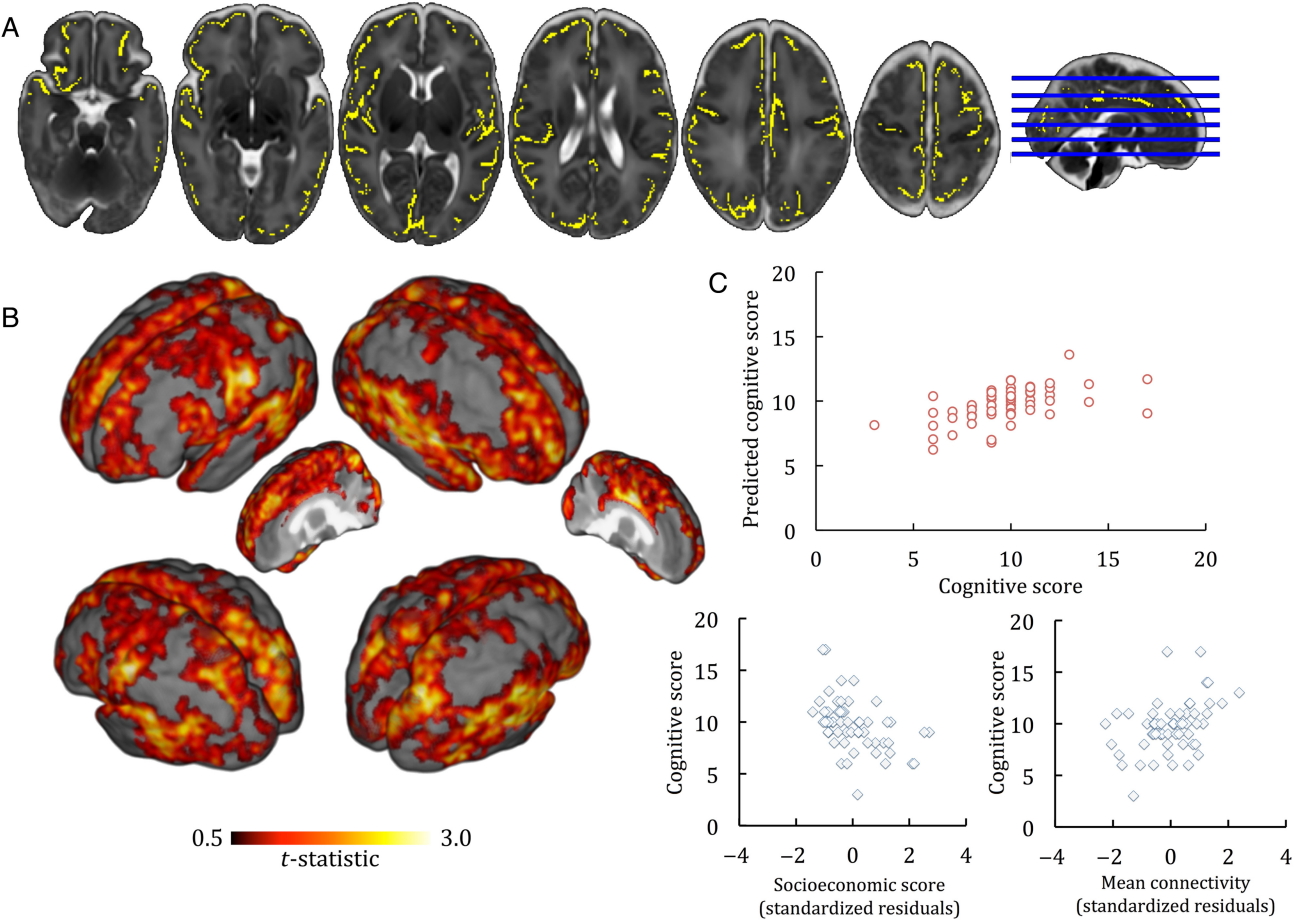
Thalamocortical connectivity and cognition. (*A*) Regions where thalamocortical connectivity at term equivalent age was correlated to cognition at 2 years, *P* < 0.05 adjusted for GA at birth, PMA at scan, and parental socioeconomic group, are shown in yellow. (*B*) Surface renderings showing the *t*-statistic of each significant voxel in A. (*C*) Full model fit (top) and semi-partial correlation plots (bottom) showing the relationship between cognitive score at 2 years, parental socioeconomic status and mean connectivity. Socioeconomic score and mean connectivity are adjusted for each other and GA at birth.

Scaled cognitive scores at 2 years were significantly positively correlated to connectivity between the thalamus and a number of cortical regions including the inferior frontal lobe, frontal pole, supplementary motor cortex, operculum, anterior and dorsal cingulum, superior parietal cortex, supramarginal gyrus, somatosensory cortex, motor cortex, superior temporal lobe, medial temporal lobe, anterior temporal lobe, and insula. Figure 1*A* shows in yellow regions where thalamocortical connectivity at term equivalent age was significantly correlated to cognition at 2 years, *P* < 0.05 adjusted for GA at birth, PMA at scan and parental SES. Figure 1*B* shows surface renderings of cortical regions where thalamocortical connectivity was significantly correlated with cognitive score at 2 years.

To establish the strength of this association, we extracted connectivity data from every voxel in the mean skeletonized cortex to enter into a multiple regression model with cognitive score, GA at birth and parental SES. This model (*F*_3,53_ = 7.57, *P* < 0.001) explained 30% of the variance in cognitive scores (*R*^2^ = 0.30; adjusted *R*^2^ = 0.26; Table 2; Fig. 1*C*). After controlling for both GA at birth and parental socioeconomic score, mean connectivity at term alone explained 10.9% of variance in cognition at 2 years of age (semi-partial [part] *r* = 0.33, *P* = 0.006; Fig. 1*C*). Parental socioeconomic score was the single largest predictor of outcome (semi-partial [part] *r* = −0.41, *P* = 0.001; Fig. 1*C*).

**Table 2:** Results of linear regression analysis of model 1

Predictors^a^	Standardized *β*	*t*	*P*	Correlations		
				Zero-order	Partial	Part
Socioeconomic score	−0.419	−3.587	0.001	−0.437	−0.442	−0.412
Mean connectivity	0.331	2.873	0.006	0.343	0.367	0.330
Gestational age at birth	0.038	0.325	0.747	0.087	0.045	0.037

^a^Overall model fit: *F*_1,53_ = 7.57, adjusted *R*^2^ = 0.26, *P* < 0.001.

**Figure 2.**
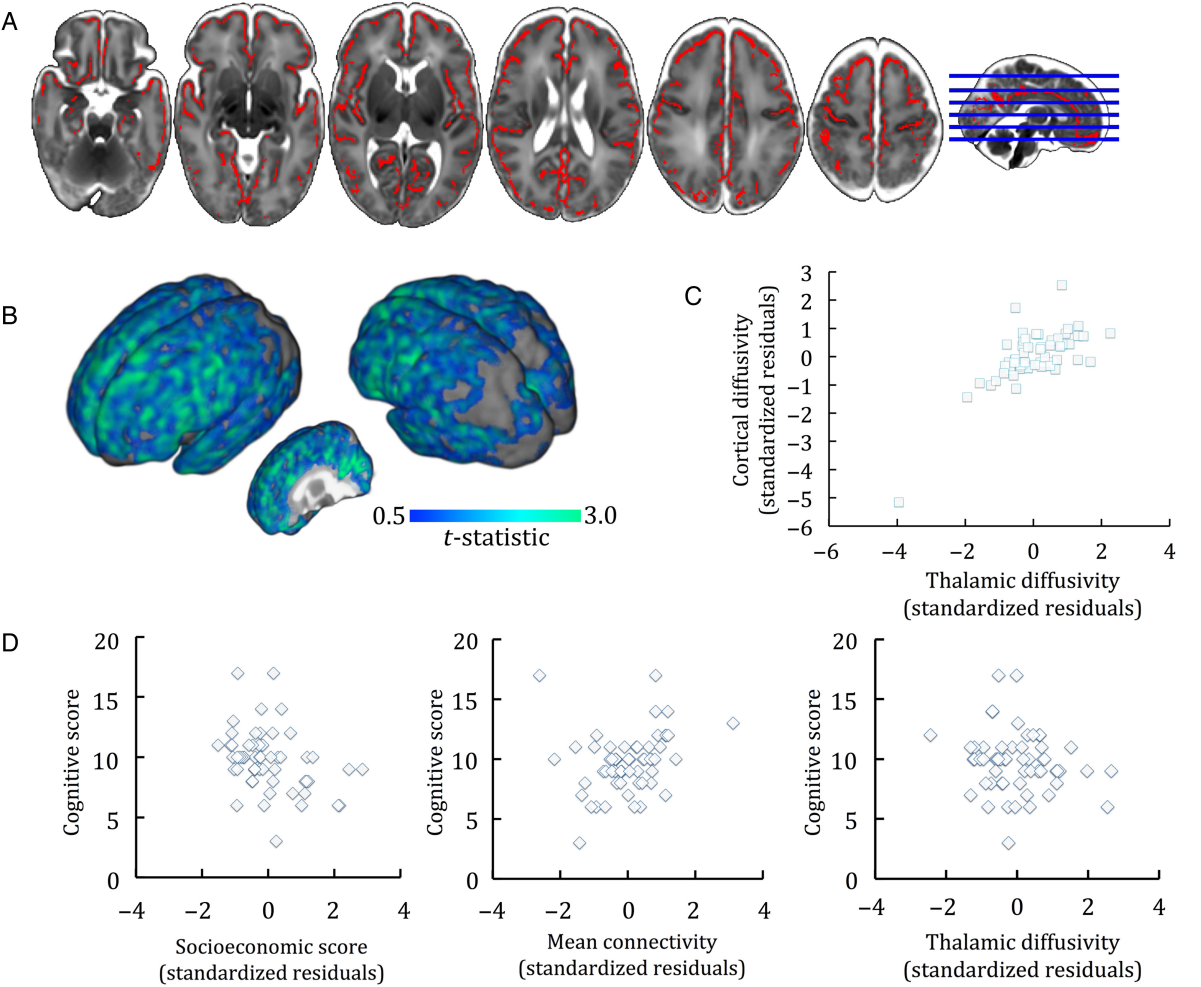
Gray matter mean diffusivity and cognition. (*A*) Regions where cortical diffusivity was correlated to cognition at 2 years, *P* < 0.01 adjusted for GA at birth, PMA at scan and parental socioeconomic group, are shown in red. (*B*) surface rendering of the t-statistic of each significant voxel in A. (*C*) correlation between mean cortical diffusivity and thalamic diffusivity, corrected for GA at birth and age at scan. (*D*) Semi-partial correlation plots showing the relationships between cognitive score, parental socioeconomic status (of note a higher SES denotes a lower socioeconomic group), mean thalamocortical connectivity and mean thalamic diffusivity.

We further examined the role of the thalamocortical system on cognitive outcome using mean diffusivity as a marker of gray matter development in the cortex and thalamus. Figure 2 shows regions where cortical mean diffusivity was significantly negatively associated with outcome, after adjusting for PMA at scan, GA at birth, and parental SES. Mean cortical diffusivity was extracted from across the cortical skeleton and entered into an explanatory model alongside thalamic diffusivity, GA at birth and parental SES (Table 3; Fig. 2*D*). This model explained 39.5% of the variance in cognitive score (*R*^2^ = 0.395; adjusted *R*^2^ = 0.336), with parental SES accounting for 11.8% (*P* = 0.003), mean thalamocortical connectivity for 5.7% (*P* = 0.033) and thalamic diffusivity for 3.0% (*P* = 0.116). Cortical diffusivity was largely dependent on thalamic diffusivity (Linear regression: thalamic diffusivity, PMA at scan, GA at birth: *R*^2^ = 0.58 [adjusted *R*^2^ = 0.55], *P* < 0.001; semi-partial *r* = 0.65, −0.17 and 0.16 respectively; Fig. 2*C*), and removing cortical diffusivity from the model did not alter the overall fit (*R*^2^ = 0.39; adjusted *R*^2^ = 0.345; *R*^2^ change = 0.004; Fig. 2*D*).

**Table 3:** Results of linear regression analysis of model 2

Predictors^a^	Standardized ß	*t*	*P*	Correlations		
				Zero-order	Partial	Part
Socioeconomic score	−0.356	−3.149	0.003	−0.437	−0.403	−0.343
Mean connectivity	0.252	2.186	0.033	0.343	0.293	0.238
Thalamic diffusivity	−0.256	−1.599	0.116	−0.431	−0.218	−0.174
Cortical diffusivity	−0.095	−0.564	0.575	−0.366	−0.079	−0.061
Gestational age at birth	0.099	0.325	0.747	0.087	0.119	0.094

^a^Overall model fit: *F*_1,51_ = 6.670, adjusted *R*^2^ = 0.34, *P* < 0.001.

## Discussion

Cognitive ability is a major predictor of important life outcomes including educational attainment, professional development and longevity (Deary 2008, 2012), although the neuroanatomical substrates associated with these performance outcomes are unclear. Preterm infants have been repeatedly shown to have worse cognitive abilities and life attainment than their term-born peers (Marlow et al. 2005; Delobel-Ayoub et al. 2009; Johnson et al. 2009) and also have widespread disruption to structural neuroanatomical architecture involving white matter, central gray, and cortical gray matter (Ajayi-Obe et al. 2000; Boardman et al. 2006; Anjari et al. 2007; Smith et al. 2011; Ball, Boardman et al. 2013; Ball, Srinivasan et al. 2013; Vinall et al. 2013; Pandit et al. 2014), thus providing an opportunity to test hypotheses concerning the neurologic substrate for complex cognitive performance.

Cognitive functions are supported by extensive networks of spatially distributed but anatomically connected brain regions (van den Heuvel et al. 2009; Tamnes et al. 2010). Intact thalamocortical connectivity in adults is essential for maintaining cognitive performance (Ystad et al. 2011; Hughes et al. 2012) and efficient information processing between brain regions relies on the integrity of white matter tracts (Penke et al. 2012). In this present study, we have shown that thalamocortical structural connectivity in the neonatal period following preterm birth is important for cognitive performance at 2 years of age.

We used a technique that we have developed to study white matter anisotropy, a surrogate marker of white matter development and structural connectivity (Robinson et al. 2010; Ball, Boardman et al. 2013), in pathways between thalamus and cortex in the neonatal brain. An advantage of this approach is that it does not rely on parcellation of the cortex into anatomically defined regions of interest a priori. We have previously demonstrated reduced connectivity between thalamus and frontal, temporal, cingulum, insula, and supplementary motor cortex in preterm infants compared with controls (Ball, Boardman et al. 2013). The present study demonstrates that impaired cognition following preterm birth is associated with impaired connectivity in distributed thalamocortical networks, including those identified previously as being disrupted following preterm birth.

Our observations extended beyond the white matter as, in addition to disrupted connectivity between thalamus and cortex, we have previously shown a dose-dependent relationship between immaturity at birth and mean diffusivity in central and cortical gray matter in preterm infants (Ball et al. 2012). Reductions in cortical diffusivity with development are thought to be associated with maturation of cortical cytoarchitecture including dendritic arborization and synapse formation (McKinstry et al. 2003; Ball, Boardman et al. 2013; Dean et al. 2013; Vinall et al. 2013). We found that lower mean diffusivity in the cortex was significantly associated with better cognitive outcome, confirming our previous findings in a separate cohort (Ball, Srinivasan et al. 2013), and demonstrating the negative impact of interruption to the shared developmental trajectory of the thalamocortical system as a whole. Cortical diffusivity and thalamic mean diffusivity were found to be strongly linked (Ball et al. 2012) and although their inclusion strengthened our model, neither independently predicted cognitive outcome when also considering thalamocortical connectivity.

Our findings are supported by neuroimaging research across the life-span; a recent longitudinal study in older children and adolescents showed that both blood oxygen level-dependent (BOLD) activity in the thalamus when performing a visuospatial working memory task and anisotropy in the surrounding white matter provide additional information regarding future cognitive performance than can be predicted by cognitive testing alone (Ullman et al. 2014), the strength of thalamic-salience network connectivity assessed by fMRI correlates with cognitive performance in early childhood (Alcauter et al 2014), and studies in adults have highlighted the involvement of distributed neural networks in cognitive processes (Tomasi et al. 2007; Charlton et al. 2010; Jefferies 2013).

In the absence of major focal lesions, preterm brain injury is characterized by abnormalities throughout the white matter that are observed on both conventional and diffusion weighted imaging at term equivalent age (Counsell et al. 2003; Anjari et al. 2007; Ball et al. 2010). These white matter abnormalities are accompanied by impaired cortical and thalamic growth at term equivalent age (Ball et al. 2012), which is associated with neurodevelopmental performance in early childhood (Boardman et al. 2010; Rathbone et al. 2011). Structural abnormalities persist into adulthood; reduced thalamic volume in adolescents born preterm is associated with decreased white matter and cortical and deep gray matter volume and these measures correlate with working memory, perceptual organization index, and processing speed (Bjuland et al. 2014); reduced cortical surface area is observed in young adults who were very low birth weight and these measures correlate with intelligence quotient scores (Skranes et al. 2013). Using network analysis of dMRI data to explore whole-brain structural connectivity, we have recently shown that the neonatal brain exhibits a “rich-club” of highly connected cortical hubs, including dorsal, medial and frontal cortex, parietal cortex, precuneus, hippocampus, and insula (Ball et al. 2014). Rich-club organization of cerebral network architecture has previously been observed in adults and is thought to represent a highly connected framework enabling efficient information processing and functional diversity (van den Heuvel and Sporns 2011; Senden et al. 2014). Preterm birth impacts on the development of the whole-brain structural connectome, resulting in reduced cortico-subcortical connectivity compared with healthy controls (Ball et al. 2014). Disruption of the thalamocortical system as a whole, therefore, is considered a major component of preterm brain injury (Volpe 2009b).

Thalamocortical connections are topographically organized and project to the majority of the cortex, acting as a link between the basal ganglia and cortex, and forming a set of parallel and segregated cortico-thalamic loops (Alexander et al. 1986; Molnar et al. 2003; Haber and Calzavara 2009). This allows thalamocortical information flow to drive ascending integration of sensory information into higher-order cortical networks (Sherman and Guillery 1998). In addition to this functional segregation, nonspecific functional afferents and nonreciprocal cortical efferents to the thalamus promote the integration and distribution of information across the cortex. It has been theorized that higher-order thalamic nuclei modulate cortico-cortical transmission via cortico-thalamocortical relays and support the formation of synchronous interareal cortical activity (Shipp 2003; Ramcharan et al. 2005; Van Essen 2005). The functional importance of a link between thalamocortical and cortico-cortical systems and their role in consciousness, attention, and cognitive performance have been discussed extensively and demonstrate the importance of thalamocortical connectivity in higher-order cognitive function (Bressler 1995; Llinas et al. 1998; Bressler and Menon 2010). It follows that the alterations in structural connectivity observed in this study may be reflected in the altered function of this system, resulting in poor cognitive performance.

Cognitive scores for the infants in this study were within the normal range. This is partly because we excluded any children with focal abnormality and we included moderately preterm infants in the study to provide a wide dynamic range for study. We used the Bayley Scales of Infant and Toddler Development III (Bayley 2006), the most recent version of the Bayley Scales, although the BSID-III assessment has been criticized for overestimating ability at 2 years (Anderson et al. 2010; Vohr et al. 2012) and mean cognitive scores for term born controls have recently been reported at >100 using this assessment tool (104 ± 11) (Serenius et al. 2013). In addition, while meta-analysis demonstrates that the mental development index derived from the Bayley I and II scales of development correlates with later cognitive function the included studies were not confined to children with normal conventional MRI brain scans (Luttikhuizen dos Santos et al. 2013), and to date no studies have assessed the predictive value of Bayley III cognitive scores. It is likely, however, that cognitive deficits are underestimated at 2 years of age and it will be important to refine the relationship between thalamocortical connectivity and cognitive abilities when the children are at school age.

We incorporated GA at birth and parental SES in our model as these are known independent predictors of cognitive ability (Greene et al. 2013; Serenius et al. 2013). Our model (GA at birth, parental SES, and mean thalamocortical connectivity at term equivalent age) explained 30% of the variance in cognition observed in these children at 2 years of age. Of note, genetic influences, which are considered to be a major component of cognitive ability, explain ∼20% of the variance in cognitive scores in early childhood (Bouchard 2004). In this study of relatively well preterm infants, GA at birth had relatively little impact on cognition, while parental SES and mean thalamocortical connectivity explained 17 and 11% of the variation in cognitive scores respectively.

In summary, we have shown that cognitive performance in early childhood following preterm birth is associated with thalamocortical connectivity. These data show that impairments in thalamocortical development can be identified as early as term equivalent age and so this approach can help identify those infants who are at risk of subsequent cognitive delay and can be used to assess the efficacy of potential treatments at an early age.

## Funding

This work was supported by the Medical Research Council (UK) (grant no: MR/K006355/1), the National Institute for Health Research Comprehensive Biomedical Research Centre at Guy's and St Thomas’ NHS Foundation Trust in partnership with King's College London and King's College Hospital NHS Foundation Trust, and Imperial College Healthcare Comprehensive Biomedical Research Centre Funding Scheme. Funding to pay the Open Access publication charges for this article was provided by the Medical Research Council (UK).
